# Human umbilical cord blood cells suffer major modification by fixatives and anticoagulants

**DOI:** 10.3389/fphys.2023.1070474

**Published:** 2023-03-15

**Authors:** Roberta Danusso, Riccardo Rosati, Luca Possenti, Elena Lombardini, Francesca Gigli, Maria Laura Costantino, Enrico Ferrazzi, Giustina Casagrande, Debora Lattuada

**Affiliations:** ^1^ Department of Women-Child-Newborn, Foundation IRCCS Ca’ Granda Ospedale Maggiore Policlinico, Milano, Italy; ^2^ Department of Medicine and Surgery, University of Milano-Bicocca, Monza, Italy; ^3^ LaBS, Department of Chemistry, Materials and Chemical Engineering “Giulio Natta”, Politecnico di Milano, Milan, Italy

**Keywords:** cell dimension, umbilical cord blood cells, fixatives, cell preservatives, anticoagulants, bio-image analysis, confocal 3D microscopy, ImageJ analysis

## Abstract

**Introduction:** Developing techniques for the tagless isolation of homogeneous cell populations in physiological-like conditions is of great interest in medical research. A particular case is Gravitational Field-Flow Fractionation (GrFFF), which can be run avoiding cell fixation, and that was already used to separate viable cells. Cell dimensions have a key role in this process. However, their dimensions under physiological-like conditions are not easily known since the most diffused measurement techniques are performed on fixed cells, and the fixation used to preserve tissues can alter the cell size. This work aims to obtain and compare cell size data under physiological-like conditions and in the presence of a fixative.

**Methods:** We developed a new protocol that allows the analysis of blood cells in different conditions. Then, we applied it to obtain a dataset of human cord blood cell dimensions from 32 subjects, comparing two tubes with anticoagulants (EDTA and Citrate) and two tubes with different preservatives (CellRescue and CellSave). We analyzed a total of 2071 cells by using confocal microscopy *via* bio-imaging to assess dimensions (cellular and nuclear) and morphology.

**Results:** Cell diameter measured does not differ when using the different anticoagulants, except for the increase reported for monocyte in the presence of citrate. Instead, cell dimensions differ when comparing anticoagulants and cell preservative tubes, with a few exceptions. Cells characterized by high cytoplasm content show a reduction in their size, while morphology appears always preserved. In a subgroup of cells, 3D reconstruction was performed. Cell and nucleus volumes were estimated using different methods (specific 3D tool or reconstruction from 2D projection).

**Discussion:** We found that some cell types benefit from a complete 3D analysis because they contain non-spherical structures (mainly for cells characterized by poly-lobated nucleus). Overall, we showed the effect of the preservatives mixture on cell dimensions. Such an effect must be considered when dealing with problems highly dependent on cell size, such as GrFFF. Additionally, such information is crucial in computational models increasingly being employed to simulate biological events.

## 1 Introduction

Nowadays, a significant challenge regards the development of simple and non-invasive methodologies for cell sorting, also considering the physiological-like and tag-less isolation of homogeneous cell populations (Roda et al., 2008; Roda et al., 2009a; Roda et al., 2009b; Lattuada et al., 2013). A particular case for tagless isolation is based on Field-Flow Fractionation (FFF) methods, which have already been used in other sectors, with few applications to biology (Giddings, 1989; Roda et al., 2009b; Radtke et al., 2015).

Cells are sorted by different features based on the nature of the field employed, resulting in several variants of these techniques, i.e., Gravitational Field-Flow Fractionation (GrFFF), Asymmetric Field-Flow Fractionation, and Electrical Field-Flow Fractionation.

For instance, the GrFFF method has already been used for the separation of lymphocyte (Roda et al., 2008), hematopoietic stem, and progenitor cells from peripheral blood (Roda et al., 2009a), and the separation of human umbilical vein endothelial cells (HUVEC) from umbilical cord blood samples (Lattuada et al., 2013). After sorting, the cells can be used for different applications such as gene expression analysis, DNA/RNA extraction, and the study of population-specific cellular processes or cell-based therapies. In these last cases, the cells had to be viable after sorting. Indeed, the FFF methods can be run avoiding cell fixation, namely, under physiological-like conditions, and they exploit morphological and physical properties of the cells for their sorting, e.g., dimension, density, and surface characteristics (Giddings, 1989; Roda et al., 2009b; Radtke et al., 2015). Remarkably, cell dimensions have a crucial role in the GrFFF sorting process (Guo et al., 2016).

However, cell dimensions under physiological-like conditions are not easily known since the standard and most diffused imaging techniques are performed on fixed cells (Mandeville et al., 1997; Graf and Boppart, 2010; Delgado-Font et al., 2020). Usually, fixation is used to rapidly and uniformly preserve tissue in a life-like state and to stabilize the fine structural details of cells and tissues before examination by light or even electron microscopy (Kiernan, 2000). In general, fixatives denature proteins *via* coagulation or cross-linking (Howat and Wilson, 2014), thereby terminating biochemical reactions. This process alters the cytoplasmic fluorescence and the cell size (Abay et al., 2019; Zhu et al., 2021).

Such alterations also regard cell dimension, affecting the separation processes designed to isolate specific cell populations (Guo et al., 2016). Indeed when using both analytical (Williams and Koch, 1992; Williams et al., 1994; Shendruk and Slater, 2012) and computational models (Jarvas and Guttman, 2013; Huang et al., 2017), the knowledge of the cellular dimension under physiological-like conditions is essential to obtain realistic results.

Therefore, this work aims to obtain and compare cell size data under two physiological-like conditions and in the presence of two different fixative mixtures.

To this end, we chose the cord blood samples as sources for this study, given the amount of blood required and the presence of all the blood cell types (including nucleated red blood cells). For this purpose, we developed a new protocol for confocal microscopy analysis under physiological-like conditions. Furthermore, by analyzing the fixed cell size, we compared the data obtained through our new protocol for confocal microscope with the data acquired by other techniques (Dinčić et al., 2021). In addition, we evaluated different methods for cell, and nuclear volume estimation from 2D analysis and 3D reconstructions obtained using different approaches.

## 2 Materials and methods

### 2.1 Study design

The analysis of cells dimension has been conducted employing two different anticoagulants, considered as physiological-like conditions (EDTA and citrate dextrose), and two different fixative mixtures containing diverse cell preservative and Polyethylene glycol (PEG) percentages. Four commercial tubes were used to collect cord blood cells for this work to increase the reproducibility of the data. We considered the most used anticoagulants and preservatives: EDTA or citrate dextrose for the first (Banfi et al., 2007; Getz et al., 2019) and two commercial solutions based on a cell preservative and PEG for the second ones. The latter differ in the cell preservatives percentage for the fixation (Stefansson et al., 2016). Therefore, we have considered four different groups characterized by different treatments: 1) EDTA, 2) citrate dextrose (CIT), 3) EDTA + 3% Cell Preservative + 0.18% PEG (CellRescue tubes—CR), 4) EDTA + 36% Cell Preservative + 0.36% PEG (CellSave tubes—CS). 32 subjects were involved in the study, collecting blood from the placentas. Among those, 22 were used to isolate cells and carry out morphological characterization, whereas 10 were for the complete blood count, i.e., a cross-comparison of the cell count for each tube.

The following protocols were applied to distinguish populations of Nucleated Red Blood Cells (NRBC), Red Blood Cells (RBC), granulocyte, lymphocyte, and monocyte.

**FIGURE 1:**
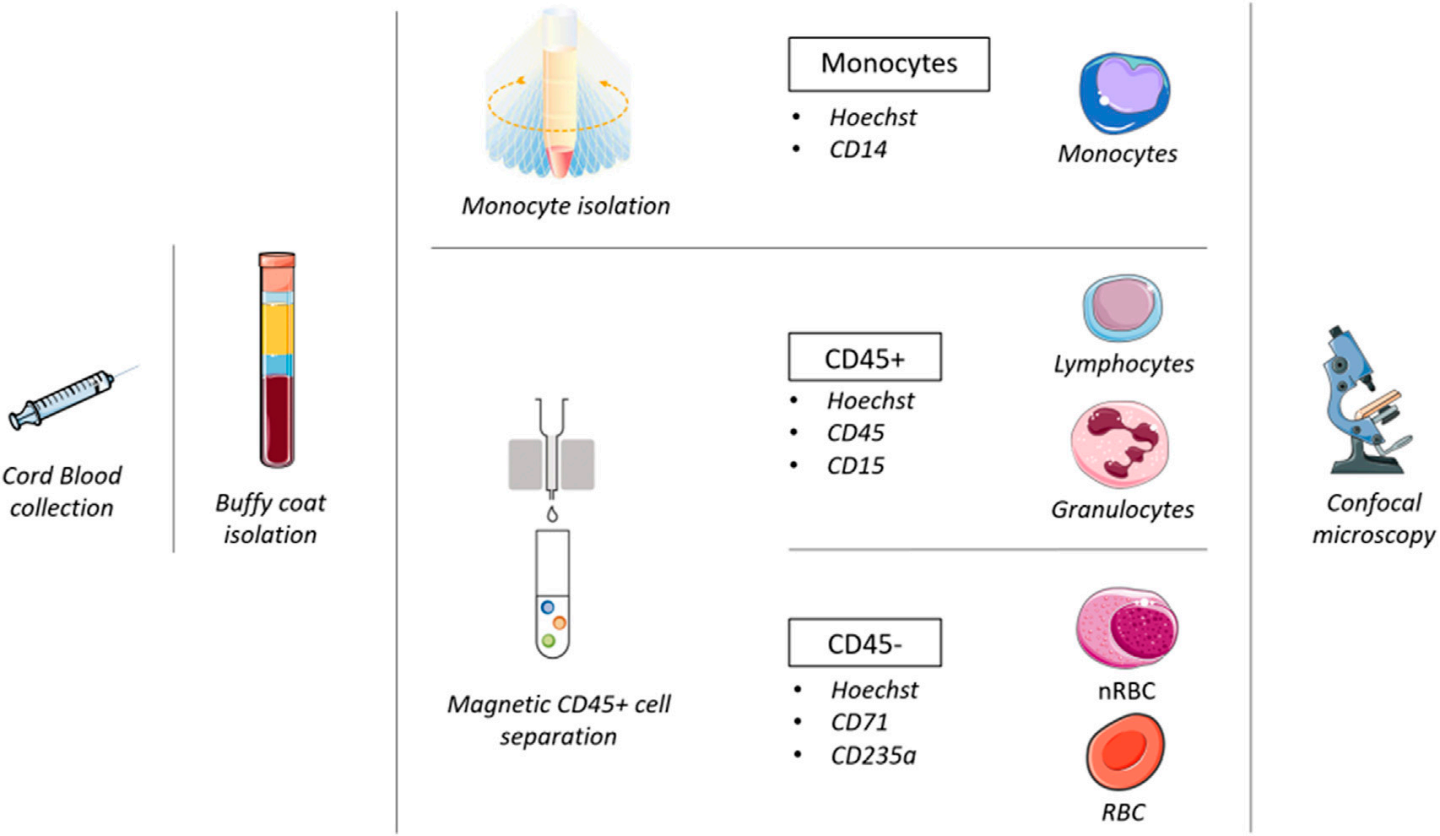
Workflow followed in this study.

Monocytes were separated with a double gradient and labeled with CD14. The other cell populations were selected by magnetic cell separation. The CD45-blood cells (NRBC and RBC) were labeled with CD71 and glycophorin, while the white cells (lymphocyte and granulocyte) were labeled with CD45+ and CD15. Figure 1 summarizes the protocol developed for this study.

We point out that basophils are not included in the granulocyte category for this work. Indeed, we used a CD15 antibody, binding the carbohydrate adhesion molecule that mediates phagocytosis and chemotaxis. Such a molecule is found on neutrophils and eosinophils but not on basophils (Satoh et al., 1994).

Cells were observed and measured by confocal microscopy after immunostaining. The dimensions were compared to evaluate the impact of treatments and the differences between different cell blood populations.

### 2.2 Subjects

This study was performed at Foundation IRCCS Ca’ Granda Ospedale Maggiore Policlinico “Department of women-child-newborn, Obstetrics and Gynaecology” in Milan. Pregnant women hospitalized for cesarean section were asked to donate their cord blood after signing informed consent approved by the Hospital Ethical Committee (Establishment n. 361 on 14/02/2012 acts n. 367/12).

Cord blood from 22 women of the 32 enrolled was used for morphometric analysis to evaluate blood cells dimension. The list of patients and the related data are reported in Table 1.

**TABLE 1:** Newborn data sex, weight (kg), APGAR score, pH, pO2, pCO2, and BE for both umbilical artery and vein.

Sex	Weight (kg)	APGAR	pH umbilical artery	Artery pCO2	Artery pO2	Artery BE	pH umbilical vein	Vein pCO2	Vein pO2	Vein BE
12 male	3.070 ± 0.510	9/10	7.27 ± 0.05	55.11 ± 12.56	10.26 ± 7.32	−0.9 ± 2.34	7.35 ± 0.05	45.5 ± 6.225	18.47 ± 9.61	−0.61 ± 1.16
19 female										

For each patient, cord blood (10 mL) was collected into vacuum tubes containing one of the four solutions detailed above (EDTA, CIT, CR, CS). Blood collected with EDTA or CIT was processed the same day within 30 min of collection, whereas blood in CR or CS tubes was processed 24 h after collection. To isolate the different cell types, we applied the same protocols regardless of the type of tube employed (see “*Cell separation and staining*” section).

For the other 10 out of the 32 women enrolled, cord blood has been collected to evaluate the cell count by complete blood count. Before treating the samples, we verified each sample to ensure that the subjects and the newborn were healthy by checking that the following indexes were in the normal range: newborn APGAR score (index of the primary function of the newborn describing heartbeats, respiration, muscle movements, reflexes, skin color), pH, partial pressure of oxygen (pO_2_), partial pressure of carbon dioxide (pCO_2_), base excess (BE) for both umbilical artery and vein.

### 2.3 Anticoagulants and cell preservatives

#### 2.3.1 Anticoagulants

Ethylenediamine tetraacetic acid (EDTA) is a polyprotic acid containing four carboxylic acid groups and two amine groups with lone-pair electrons chelating calcium and many other metal ions. Several enzymatic reactions composing the coagulation cascade occur due to the presence of calcium. Therefore its removal can irreversibly avoid blood clotting in the tube. In the field of *in-vitro* diagnostics, EDTA is recommended as the anticoagulant of choice for hematological analysis because it preserves cellular components and the morphology of blood cells for a long time (Banfi et al., 2007). In particular, Ethylenediaminetetraacetic Acid Tripotassium Dihydrate (K_3_EDTA) (Vacuette) tubes have been used to collect blood.

Anticoagulant-Citrate-Dextrose (CIT) consists of Trisodium Citrate, 22.0 g/L, Citric Acid, 8.0 g/L, and Dextrose, 24.5 g/L. It is commonly used as an anticoagulant (BD Vacutainer) to preserve blood samples required for tissue typing, ESR measurement, the study of coagulation factors (e.g., fibrinogen, PT, APTT) (Banfi et al., 2007), and platelet function. In addition, as it is non-toxic, it is also used as an alternative to heparin during procedures such as plasmapheresis.

#### 2.3.2 Cell preservatives

The commercial products (Menarini Silicon Biosystem, Singapore) CellRescue (CR) and CellSave (CS) were used. They contain a different percentage of cell preservatives and PEG. The CellRescue Preservative tubes contain 660 μL of 2.3% Na_2_EDTA and 3% cell preservative, 0.18% PEG, and 0.23% inert ingredients. The CellSave Preservative tubes contain 300 μL solution made of 4.6% Na_2_EDTA and 36% cell preservative, 0.36% polyethylene-glycol, and 0.46% inert ingredients.

#### 2.3.3 Measurement of osmolality in anticoagulants and cell preservatives tubes

Dulbecco’s Phosphate Buffered Saline (PBS) osmolality is close to plasma (275–290 mOsm/kg) (Gagné, 2014). PBS was added to the four tubes until they were filled for osmolality measurements. Before each measurement, the osmometer (MIR 300-M, Emanuele Mires, Italy) was checked for zero display with distilled water.

### 2.4 Buffy coat isolation

10 mL of human cord blood was mixed 1:1 with PBS without Calcium chloride and Magnesium chloride (Merck KGaA, Germany) and gently transferred into a tube containing 10 mL of Lympholyte (Euroclone, Italy) and centrifuged (780 rcf for 20’, brake 0 at RT). The buffy coat containing nucleated cells was collected and washed twice with PBS. The sample was lysed with 10 mL of RBC lysing solution containing 15.5% NH_4_Cl 1 M, 1% KHCO_3_ 1 M, and 1% EDTA 10 mM, and it was then washed with PBS.

### 2.5 Monocyte isolation

The low presence of monocyte in the blood required 5X the usual volume for the isolation. Therefore, the initial volume was 50 mL. Cord blood was collected, and the buffy coat was isolated following the protocol above. The buffy coat containing nucleated cells was washed with PBS.

The pellet was suspended in 12 mL of Modified Eagles Medium (MEM) 199 supplemented with 15% Fetal Bovine Serum, 8% L-Glutammine, and 2% Penicillin-Streptomycin. These 12 mL were divided into four parts, and each part was gently transferred into a tube containing 10 mL of hyperosmotic Percoll solution [48.5% Percoll (Merck KGaA, Germany), 41.5% H_2_O, 10% NaCl 1.6 M]. The sample was centrifuged (580 rcf for 15’, brake 0 at RT), and the layer of cells at the interface was collected, washed with PBS, lysed with 10 mL of RBC lysing solution containing 15.5% of NH_4_Cl 1 M, 1% KHCO_3_ 1 M, 1% EDTA 10 mM, and washed with PBS.

### 2.6 Cell separation and staining

Following the manufacturer’s instructions, we ran CD45 separating column (MS Columns, Miltenyi Biotec, Germany) for 10 × 10^6^ cells from the buffy coat.

After the magnetic separation, all samples were stained with 6 μL Hoechst 33,345 trihydrochloridate trihydrate (Invitrogen, MA, United States) and incubated for 20 min at 37°C.

After Hoechst staining, 3 × 10^5^ CD45+ cells were labeled with 3 μL of CD45 anti-human APC clone 5B1 (Miltenyi Biotec, Germany) and 2 μL of CD15 anti-human FITC clone VIMC6 (Miltenyi Biotec, Germany) fluorescent antibodies. 3 × 10^5^ CD45-cells were labeled with 2 μL of CD71 anti-human PE Clone AC102 (Miltenyi Biotec, Germany) and with CD235a Glycophorin A anti-human BB515 Clone GA-R2 (HIR2) (Bioscience BD, NJ, United States) fluorescent antibodies.

Instead, monocytes were labeled with 2 μL of CD14 anti-human PE-Vio clone REA599 (Miltenyi Biotec, Germany).

After 30 min of incubation, cells were centrifuged at 500 rcf for 5’ at RT and resuspended in 60 μL of PBS (Merck KGaA, Germany).

### 2.7 Confocal microscopy

The cells were observed using a White Light Laser Leica Scanning Confocal Microscope (LEICA DMi8) (×63 OIL objective, LEICA). A 15 μL cell suspension (5 × 10^6^ cells/mL) was placed over a 50 × 18 mm coverslip previously washed with methanol to remove halos or dirt. Every 15 min, 60 μL of PBS was added to the cell suspension on the coverslip to avoid dehydration and keep the solution’s correct osmolality. The acquisition resolution was set at 1024 × 1024 pixels, corresponding to a square image with a side length of 184.7 μm, and a 4x zoom was chosen to isolate the cells from the original image. The cells were acquired along their z-axis with a size step of 0.5 μm.

### 2.8 Cell count

Complete blood count was performed on the cord blood of the 10 patients using a Sysmex XN-10TM Automated Hematology Analyzer (Dasit, Italy). All four tubes (EDTA, CIT, CR, and CS) have been tested for every patient.

### 2.9 Bio-image analysis

A total of 2071 cells were analyzed *via* bio-imaging after the cell isolation processes. Even if a similar number of cells could have been isolated from a single patient, we chose to consider samples derived from different patients to increase the significance of the data. The composition of the entire sample is reported in Table 2.

**TABLE 2:** Number of collected cells for each condition. Percentages refer to the total amount of cells analyzed.

Number of cells analyzed	EDTA	CIT	CR	CS	Total cells analyzed
%Tubes	(44.4%)	(15.6%)	(11.1%)	(28.9%)	
NRBC	114	65	68	69	316 (15%)
RBC	53	31	43	220	347 (17%)
Granulocyte	236	60	51	141	488 (24%)
Lymphocyte	288	125	77	152	642 (31%)
Monocyte	115	77	45	41	278 (13%)
Total Cells collected	806	358	284	623	2071 (100%)

All images acquired were processed employing Fiji software (Schindelin et al., 2012). An *ad hoc* code was developed *via* the ImageJ Macro feature to enhance the reproducibility of the analysis. The workflow of the bio-image analysis started with assembling an image joining all the fluorescence channels (maximum intensity projection area) to perform an antigen positivity classification. Then, the particle analysis phase concluded the macro. The Supplementary Material describe all the steps to import the image (.lif file), improve quality, and reduce noise to perform an accurate analysis. We evaluated parameters such as nuclei and cells’ diameters, the percentage of nucleus area over the whole cell area, and the shape descriptors (circularity, aspect ratio, solidity) both for the cell and nucleus.

In addition, three-dimensional reconstruction was conducted for nuclei and cells employing Fiji 3DSuite Plugin (Ollion et al., 2013). For this preliminary evaluation, 40 randomly chosen suitable image sets (called stacks) were considered (Table 3). Specifically, a comparison was made between 2D orthogonal plane projections, i.e., along the X, Y, and Z axes. Then, we measured and compared cells using volume data or projection from the three cartesian axes. The selected cells’ volume and nuclei were measured in three different methods. The first (“3DSuite”) used the ImageJ 3DSuite Plugin, which provides a three-dimensional reconstruction of the cell and its nucleus. Moreover, it measures the volume, surface, and sphericity (S_f_) parameter, which measures how much an object’s shape resembles a perfect sphere. The commonly used sphericity definition first proposed by Wadell (Wadell, 1932) is the ratio of the nominal surface area (surface area of a sphere having the same volume as the object) to the actual surface area of the object:

$$S_{f}=\frac{\pi^{\frac{1}{3}}{\left(6V_{p}\right)}^{\frac{2}{3}}}{S_{p}}$$

**TABLE 3:** List of cells undergoing 3D reconstruction, subdivided by cell type and tube.

3D reconstruction	EDTA	CIT	CR	CS	TOT
NRBC	7	5	4	2	18
Granulocyte	3	6	1	1	11
Lymphocyte	4	4	1	2	11
TOT	14	15	6	5	40

where V_p_ is the particle volume, and S_p_ is the particle surface area. We point out that Fiji produces two different values for this index. We selected the ‘Corrected sphericity’, which considers the surface obtained *via* pixel interpolation. The second method (“2DProjXY”) recomposed the original stack along the *x* and *y* direction by the command “Reslice [\]…” of Fiji. Then, it computes the 2D x- and y-projection area and estimates the radius. Finally, the volume was evaluated, assuming a spherical shape. The third (“2DProjZ”) measured the area of the 2D z-projection of the original stack image and calculated the volume with the same procedure.

### 2.10 Statistical analysis

2D descriptive statistics concerns the following parameters:

● The nuclei and cell diameters;
● The percentage of nucleus area over the whole cell area;
● The shape descriptors (or factors) both for cell and nucleus:
○ Circularity: it is a value between 0 and 1 (1 = perfect circle): $4\pi \frac{Area}{Perimeter^{2}}$

○ Aspect Ratio (AR): is the ratio between the major and the minor axes of the particle’s fitted ellipse (by definition higher than 1, with 1 in case of a perfect circle);
○ Solidity: is a value between 0 and 1, and it is the ratio between the area and the convex shape area, namely, the convex region that contains the particle.

For the subset of 3D reconstructed cells, the nucleus and cell volume were also considered, as well as corrected sphericity.

The analysis has been performed employing “Statistical Package for Social Science 18.0” (SPSS Inc., Chicago, Illinois). Chi-Squared Tests have been conducted to evaluate the normal distribution of the data. In addition, the difference between groups was compared using the non-parametric Mann-Whitney U test for non-normally distributed data and the Anova test for normally distributed data. Cells’ measures are expressed as median and first and third quartile. *p*-value lower than 0.05 has been considered statistically significant (*).

## 3 Results

A total of 2071 cord blood cells were analyzed under two physiological conditions and in the presence of two fixative mixtures. Therefore, we have considered 4 different groups characterized by different treatments: 1) EDTA, 2) citrate dextrose (CIT), 3) EDTA + 3% Cell Preservative + 0.18% PEG (CellSave—CS), 4) EDTA + 36% Cell Preservative + 0.36% PEG (CellRescue—CR). Here we report the analysis results varying the four conditions starting from the 2D morphological analysis. Then we analyze the cell count and compare 2D and 3D analysis considering volumetric data.

**FIGURE 2:**
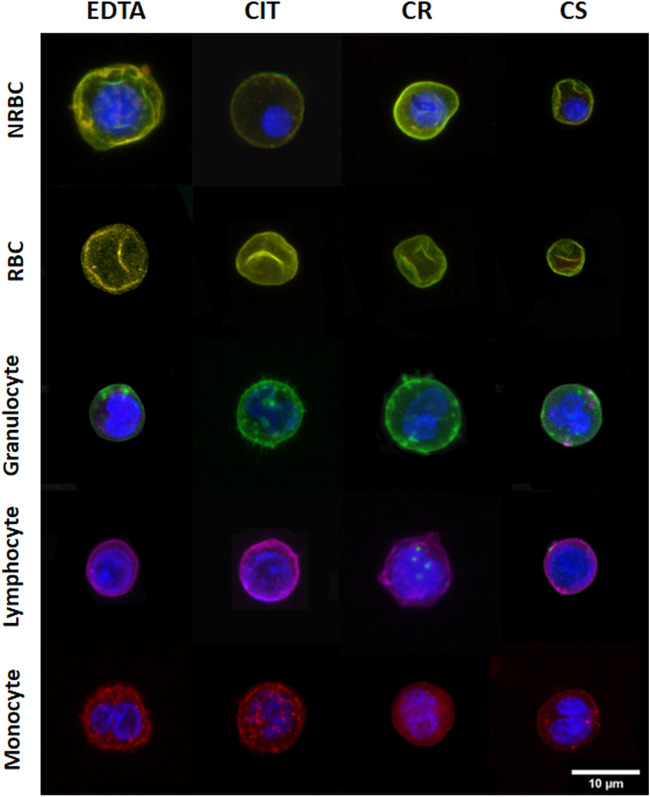
Representative 2D images of different cord blood cell groups collected in different conditions. The rows indicate the cell populations, and the columns indicate the collecting tubes.

Figure 2 shows a representative panel of 2D images of cells and nuclei in different cord blood cell populations under the four conditions analyzed.

### 3.1 Bidimensional morphological analysis

#### 3.1.1 Cellular diameter

**FIGURE 3:**
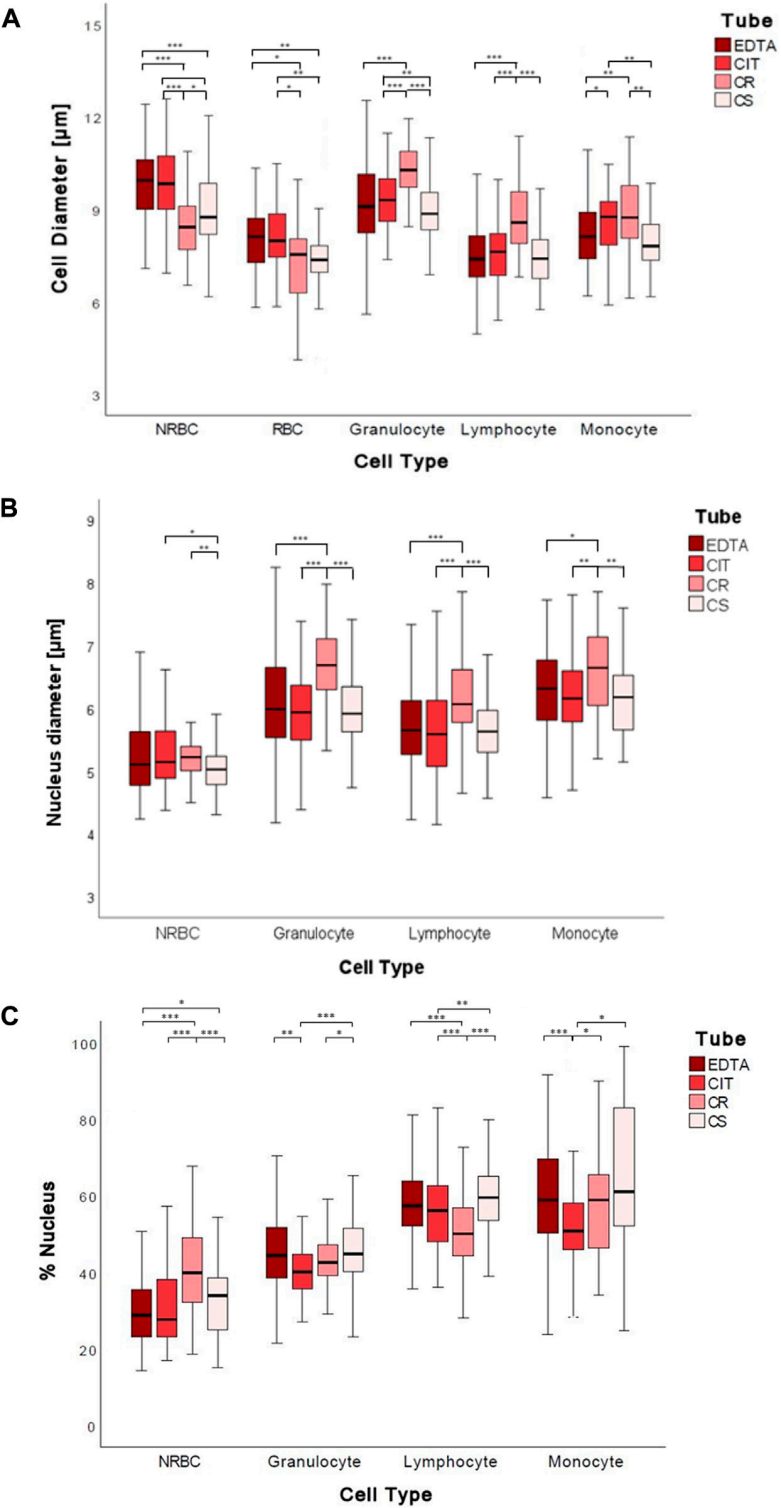
Boxplots of 2D cell diameters **(A)**, nuclear diameters **(B)**, and percentage of the nucleus **(C)** measured in µm for each tube for each cell group. The symbol * represents a statistically significant difference (*p < 0.05; **p < 0.01; ***p < 0.001).

Significant changes were found in the diameter of cord blood cells [NRBC, RBC, granulocyte (G), lymphocyte (L), and monocyte (M)] collected in the 4 different conditions analyzed. In particular, the medians ranges NRBC 8.44–9.95 µm, RBC 7.38–8.13 µm, granulocyte 8.86–10.29 µm, lymphocyte 7.4–8.58 µm, monocyte 7.81–8.76 µm (Figure 3A; Table 4).

**TABLE 4:** Cell diameters, nucleus diameters, and nucleus percentages for each cell group for each tube. The data are reported as median (1st–3rd quartile).

Cell diameter				
	EDTA	CIT	CR	CS
NRBC	9.95 (9.01–10.63)	8.85 (8.94–10.75)*§*	8.44 (7.7–9.11)*§*	8.76 (8.17–9.92)*§*
RBC	8.13 (7.24–8.76)*§*	7.99 (7.43–8.87)*§*	7.55 (6.02–8.08)	7.38 (6.97–7.83)
Granulocyte	9.1 (8.26–10.14)	9.31 (8.62–10.01)*§*	10.29 (9.69–10.89)*§*	8.86 (8.35–9.57)*§*
Lymphocyte	7.4 (6.82–8.16)	7.64 (6.86–8.24)	8.58 (7.89–9.6)*§*	7.98 (7.42–8.75)*§*
Monocyte	8.13 (7.41–8.94)	8.76 (7.85–9.27)	8.74 (8.05–9.78)*§*	7.81 (7.35–8.53)*§*
Nucleus diameter				
	EDTA	CIT	CR	CS
NRBC	5.11 (4.77–5.63)	5.15 (4.88–5.63)	5.22 (5.01–5.39)	5.03 (4.77–5.24)
Granulocyte	5.99 (5.54–6.65)*§*	5.94 (5.48–6.41)*§*	6.69 (6.29–7.11)*§*	5.92 (5.62–6.35)
Lymphocyte	5.6 (5.27–6.13)	5.59 (5.07–6.13)	6.0 (5.77–6.62)	5.64 (5.30–5.97)
Monocyte	6.32 (5.77–6.78)	6.16 (5.76–6.61)*§*	6.65 (5.97–7.15)*§*	6.18 (5.65–6.53)*§*
Nucleus percentage				
	EDTA	CIT	CR	CS
NRBC	28.84 (23.10–36.06)	27.76 (22.90–38.70)	39.95 (31.84–49.02)*§*	33.99 (25.00–39.54)*§*
Granulocyte	44.48 (38.54–51.79)*§*	40.14 (35.74–44.82)*§*	42.61 (39.05–47.80)*§*	44.88 (40.23–51.86)
Lymphocyte	51.17 (51.94–63.47)*§*	55.23 (47.87–62.20)*§*	50.17 (44.29–56.85)*§*	59.50 (53.24–64.84)*§*
Monocyte	57.23 (50.07–68.69)*§*	50.60 (45.48–57.73)*§*	58.48 (45.91–64.63)*§*	55.43 (48.85–70.39)*§*

The symbol § indicates that the data are normally distributed.

By analyzing the diameter of cord blood cells, non-statistically significant differences are reported between cells from human cord blood collected in EDTA and CIT, except for monocyte, whose dimensions are increased in CIT (*p* = 0.017). Instead, when comparing anticoagulants and cell preservative tubes, we often have a statistically significant difference in cell dimensions, with a few exceptions [5 out of 30 combinations: granulocyte EDTA vs. CS (*p* = 0.236); lymphocyte EDTA vs. CS (*p* = 0.431) and CIT vs. CS (*p* = 0.079) and monocyte EDTA vs. CS (*p* = 0.388) and CIT vs. CR (*p* = 0.182)] (Supplementary Table S2).

Interestingly, the four conditions have different effects on the cells. Indeed, by ordering the cell types by the cell diameter, we obtain four different lists (EDTA: *NRBC, G, RBC/M, L*; CIT: *G, NRBC, M, RBC, L*; CR: *G, M, L, NRBC, RBC*; CS: *G, NRBC, L, M, RBC*).

Comparing conditions with and without preservatives, we cannot identify a common path of change; namely, not all the cells are smaller or larger when fixed (e.g., NRBC with CR are smaller than with EDTA, whereas granulocyte with CR are larger than with EDTA).

The maximum difference in cell diameter, for each cell type, in the four conditions ranges between 0.75 and 1.43 µm, i.e., roughly 10% of the cell diameter.

#### 3.1.2 Nuclear diameter

In most cases, the anticoagulant (EDTA, CIT) and CS conditions have no different effects on the nucleus diameter of cells from human cord blood; instead, CR shows a significant increase in all white blood cells (G, L, M). The nuclear diameter medians were for NRBC 5.03–5.22 µm, granulocyte 5.92–6.69 µm, lymphocyte 5.64–6.07 µm, and monocyte 6.16–6.65 µm (Figure 3B; Table 4).

In detail, nuclear diameter is significantly increased in granulocyte CR vs. EDTA (*p* < 0.001), CR vs. CIT (*p* < 0.001), CR vs. CS (*p* < 0.001); lymphocyte CR vs. EDTA (*p* < 0.001), CR vs. CIT (*p* < 0.001), CR vs. CS (*p* < 0.001); and monocyte CR vs. EDTA (*p* = 0.019), CR vs. CIT (*p* = 0.002), CR vs. CS (*p* = 0.003). In NRBC, the nuclear diameter is significantly increased in CR vs. CS (*p* = 0.001) and significantly decreased in CS vs. CIT (*p* = 0.027) (Supplementary Table S2).

The maximum difference in nuclear diameter for each cell type in the four conditions tested ranges between 0.19 and 0.77 μm, 8% of the nuclear diameter on average.

#### 3.1.3 Nuclear percentage in cells

We also analyzed changes in the percentages of the nucleus, namely, the area of the nucleus over the area of the whole cell. In particular, the median percentages of the nucleus were for NRBC 27.8%–40.0%, granulocyte 40.1%–44.9%, lymphocyte 50.2%–59.5%, and monocyte 50.6%–58.5% (Figure 3C; Table 4).

The percentage of the nucleus is often similar when comparing the anticoagulants except for a decrease for the CIT vs. EDTA granulocyte (*p* < 0.001) and monocyte case (*p* < 0.001) (Supplementary Table S2). Conversely, a significant difference is observed in the presence of fixatives. The percentage of the nucleus is significantly increased in NRBC CS vs. EDTA (*p* = 0.018), CR vs. EDTA (*p* < 0.001), and CS vs. CIT (*p* = 0.140); in granulocyte, lymphocyte, and monocyte CS vs. CIT (respectively *p* < 0.001, *p* = 0.004, and *p* = 0.011); in monocyte CR vs. CIT (*p* = 0.047). The percentage of the nucleus is significantly decreased in lymphocyte between CR vs. EDTA (*p* < 0.001), CR vs. CIT (*p* < 0.001); in monocyte CIT vs. EDTA (*p* < 0.001); Comparing the two fixatives (CS vs. CR), the percentage of the nucleus is increased in granulocyte, and lymphocyte and decreased in NRBC (respectively *p* = 0.026, *p* < 0.001, and *p* < 0.001) (Supplementary Table S2).

The largest variation considering anticoagulants vs. preservatives is found with NRBC. Indeed, they are different from the other cells under physiological-like conditions and closer in size to the other cell types when treated with cell preservatives.

#### 3.1.4 Shape descriptors

**FIGURE 4:**
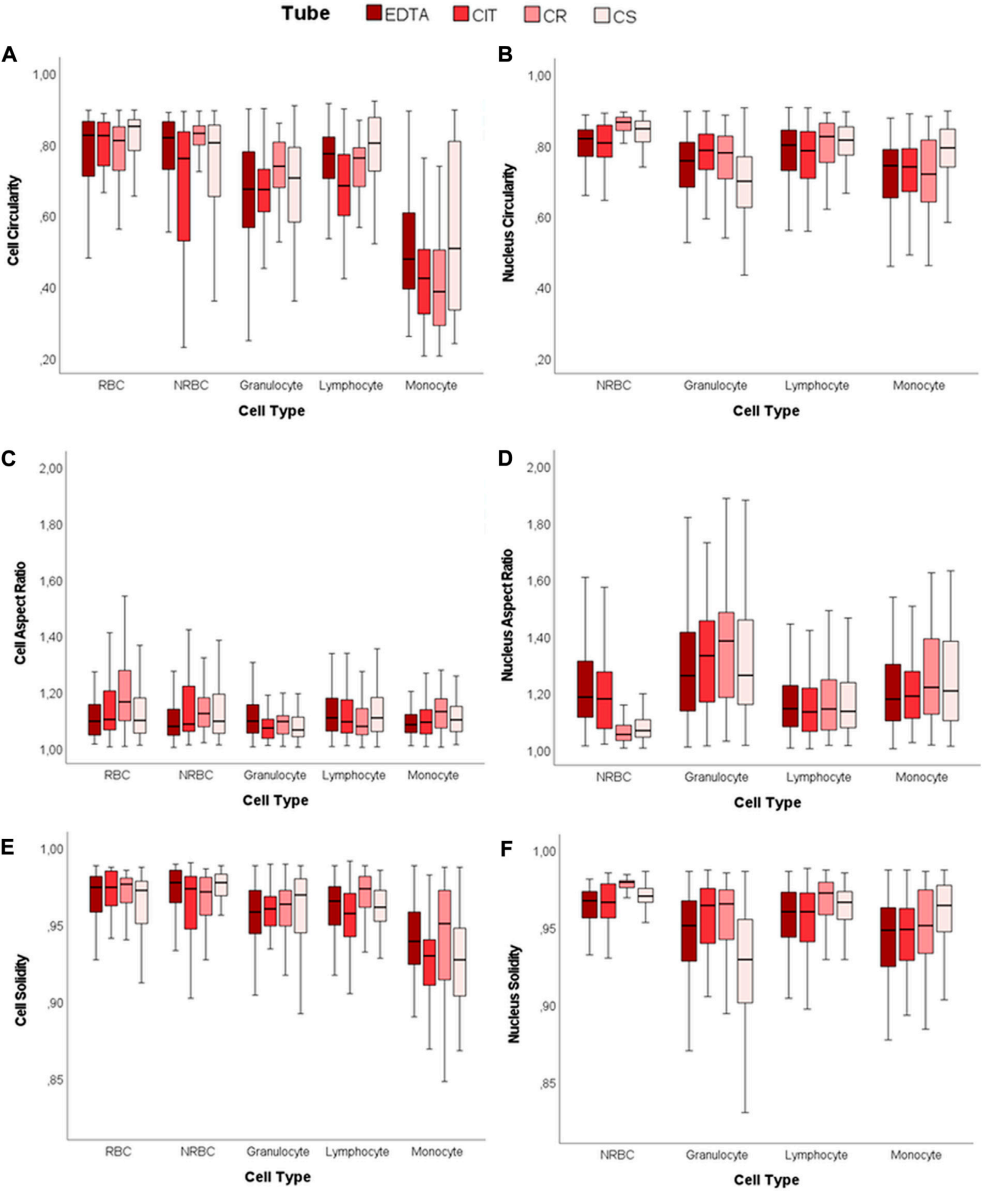
2D shape descriptors. Boxplots of cell and nucleus circularity, aspect ratio, and solidity. **(A)** Cell Circularity, **(B)** Nucleus Circularity, **(C)** Cell Aspect Ratio, **(D)** Nucleus Aspect Ratio, **(E)** Cell Solidity, and **(F)** Nucleus Solidity.

Circularity, Aspect Ratio (AR), and Solidity are the parameters considered for determining shape descriptors of both the cell and nuclei contours for each cell type and each tube (Figure 4; Supplementary Tables S3–S6). Overall, even if there are some differences in the numerical value of the indexes, these data show no relevant alterations in the four cases considered.

For each tube, the cell and nucleus circularity median values approach unity, denoting a shape close to a perfect circle for both the cell and the nucleus. The cell circularity values are similar for NRBC and RBC (CIT is the only significant difference out of the 4 conditions). However, white blood cells (WBCs) have lower cell and nucleus circularity values than NRBC and RBC (cell circularity significantly different in 6/6 cases for EDTA, 5/6 in CIT, 6/6 in CR, 5/6 in CS; nucleus circularity reaches significance on 3/3 cases under all the four conditions). The AR data also support these results.

Nuclear AR of lymphocyte and NRBC indicate that the nucleus is closer to a circle than in the other two cell populations. This value deviates more from unity in granulocyte (lymphocyte vs. granulocyte *p* < 0.001, NRBC vs. granulocyte *p* < 0.001, for all four tubes), as expected, since they have a poly-lobate nucleus.

The solidity highlights if the object is characterized by external or internal irregularities (e.g., indentations, peaks, or holes). For each tube and cell type, all the values of cells and nuclei are in the range of 0.93–0.98, very close to unity. Although some statistical difference appears, they are related to minor differences in median values. The variability reported for granulocyte might be related to nuclear poly-lobality.

#### 3.1.5 Anticoagulants and cell preservatives osmolality

Given the differences among the 4 solutions, particularly in cell dimensions, we assessed the osmolality of the collecting solutions. The osmolality results reveal that anticoagulant solutions with PBS have osmolality closer to plasma than preservative solutions (PBS 299 mOsm/kg; EDTA 312 mOsm/kg; CIT 313 mOsm/kg, CR 337 mOsm/kg, CS 335 mOsm/kg, plasma 275–290 mOsm/kg).

### 3.2 Cell count

**FIGURE 5:**
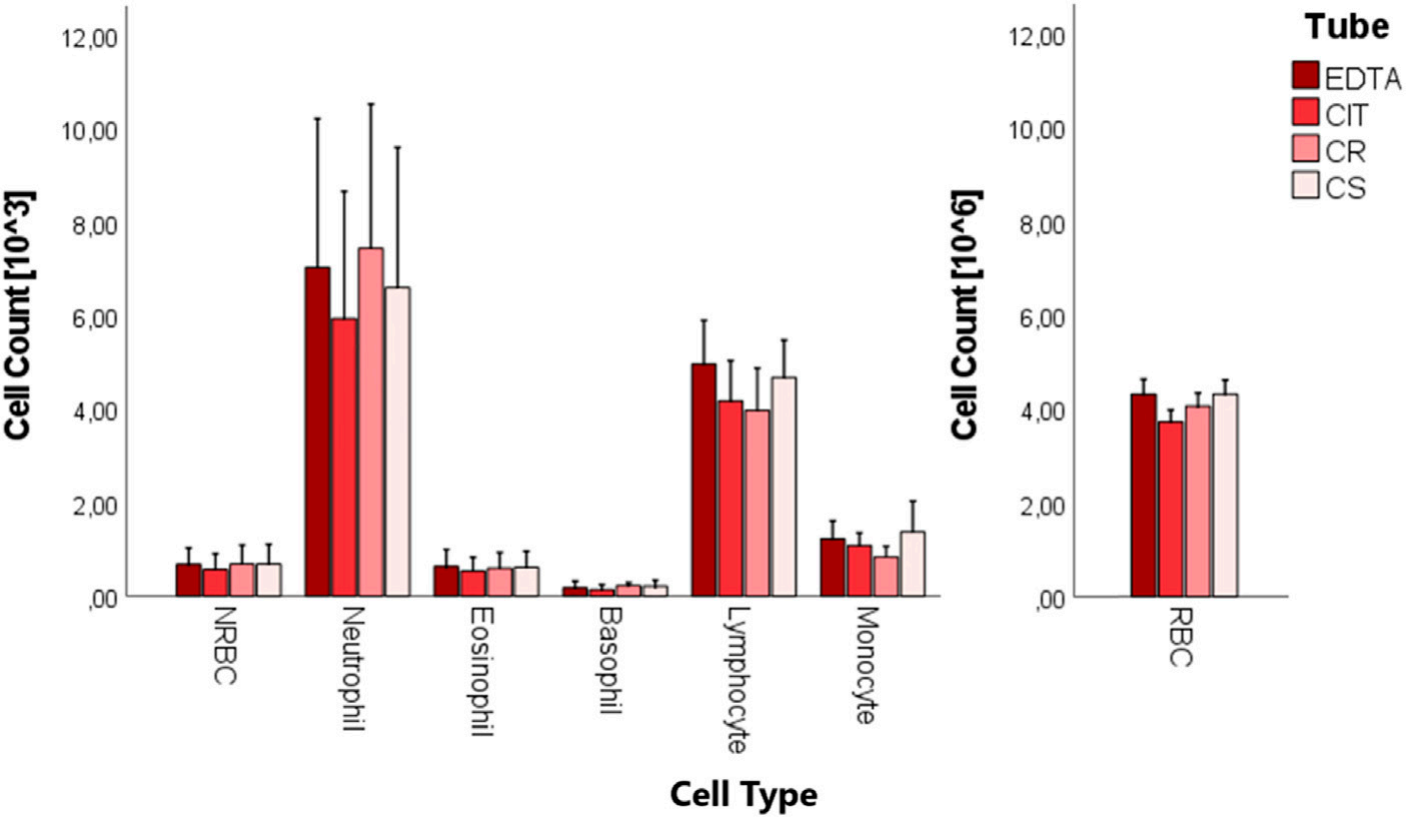
Boxplots of cord blood complete blood count analysis in the four tubes (EDTA, CIT, CR, and CS).

A complete blood count was performed on the whole cord blood of 10 patients in the four different conditions (Figure 5; Table 5). No statistically significant difference was found in the cell counts of different cell populations among the four conditions.

**TABLE 5:** Cell count [10^3^/µL] for each cell group and tube. Exclusively for RBC, the count is multiplied by 10^6^/µL. The data are reported as media ± SD.

Cell type	N	EDTA	CIT	CR	CS
NRBC [10^3/µL]	10	0.682 ± 0.352 *§*	0.570 ± 0.338 *§*	0.685 ± 0.425 *§*	0.684 ± 0.408 *§*
RBC [x10^6/µL]	10	4.251 ± 0.313 *§*	3.669 ± 0.248 *§*	4.251 ± 0.297 *§*	3.999 ± 0.272 *§*
Neutrophile [10^3/µL]	10	7.036 ± 3.181 *§*	5.943 ± 2.719 *§*	6.603 ± 3.000 *§*	7.447 ± 3.082 *§*
Eosinophile [10^3/µL]	10	0.520 ± 0.336 *§*	0.537 ± 0.295 *§*	0.616 ± 0.346 *§*	0.596 ± 0.338 *§*
Basophile [10^3/µL]	10	0.179 ± 0.142 *§*	0.135 ± 0.110 *§*	0.214 ± 0.126 *§*	0.223 ± 0.063 *§*
Lymphocyte [10^3/µL]	10	4.966 ± 0.937 *§*	4.178 ± 0.862 *§*	4.681 ± 0.799 *§*	3.975 ± 0.905 *§*
Monocyte [10^3/µL]	10	1.225 ± 0.385 *§*	1.083 ± 0.272 *§*	1.374 ± 0.657 *§*	0.836 ± 0.227 *§*

The symbol § indicates that the data are normally distributed.

### 3.3 Tridimensional volume data analysis

**FIGURE 6:**
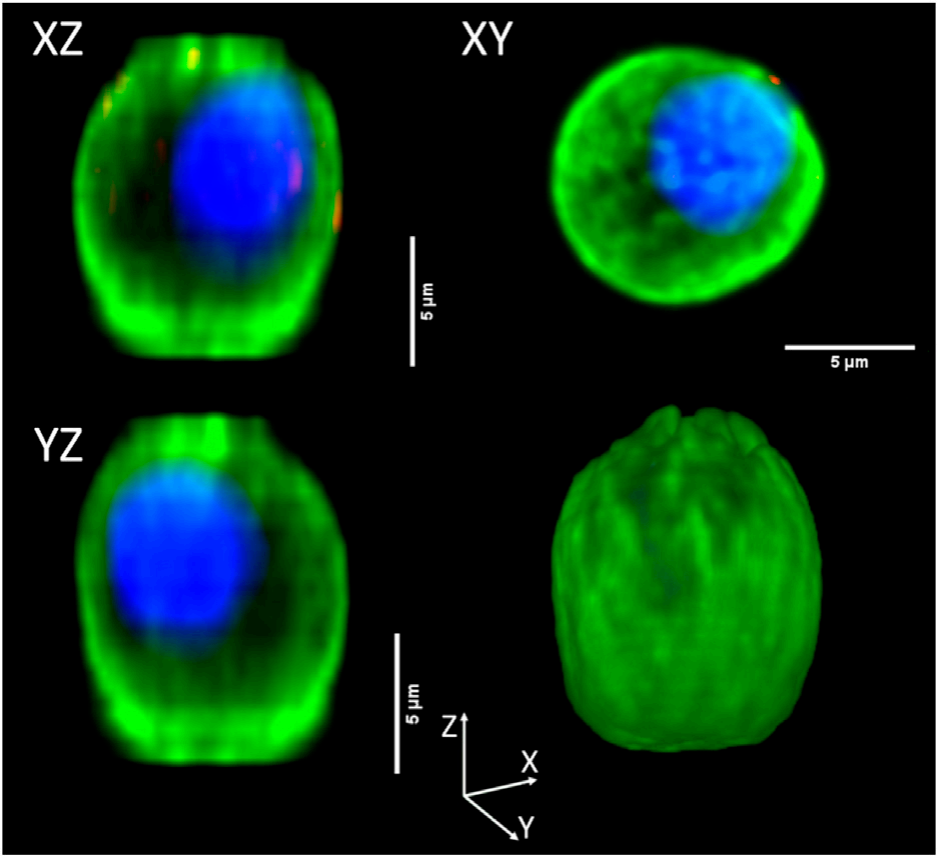
Orthogonal view of a three-dimensional reconstructed NRBC. The top and bottom left corner panels, namely, “XZ” and “YZ,” are the projections along Y and X orthogonal views, respectively (“2DProjXY” method), the top right corner (“XY”) is the projection along the Z axis (“2DProjZ” method). The bottom right corner panel shows the 3D reconstruction (“3DSuite” method).

For a preliminary evaluation, we selected 40 cord blood cells in which reconstruct, measure, and compare cell and nuclear volumes obtained *via* different measurement methods: “3DSuite” by ImageJ PlugIn, “2DProjXY” and “2DProjZ” by 2D orthogonal plane projections. As an example of the three different methods for 3D reconstruction, Figure 6 shows the orthogonal views of an NRBC.

**FIGURE 7:**
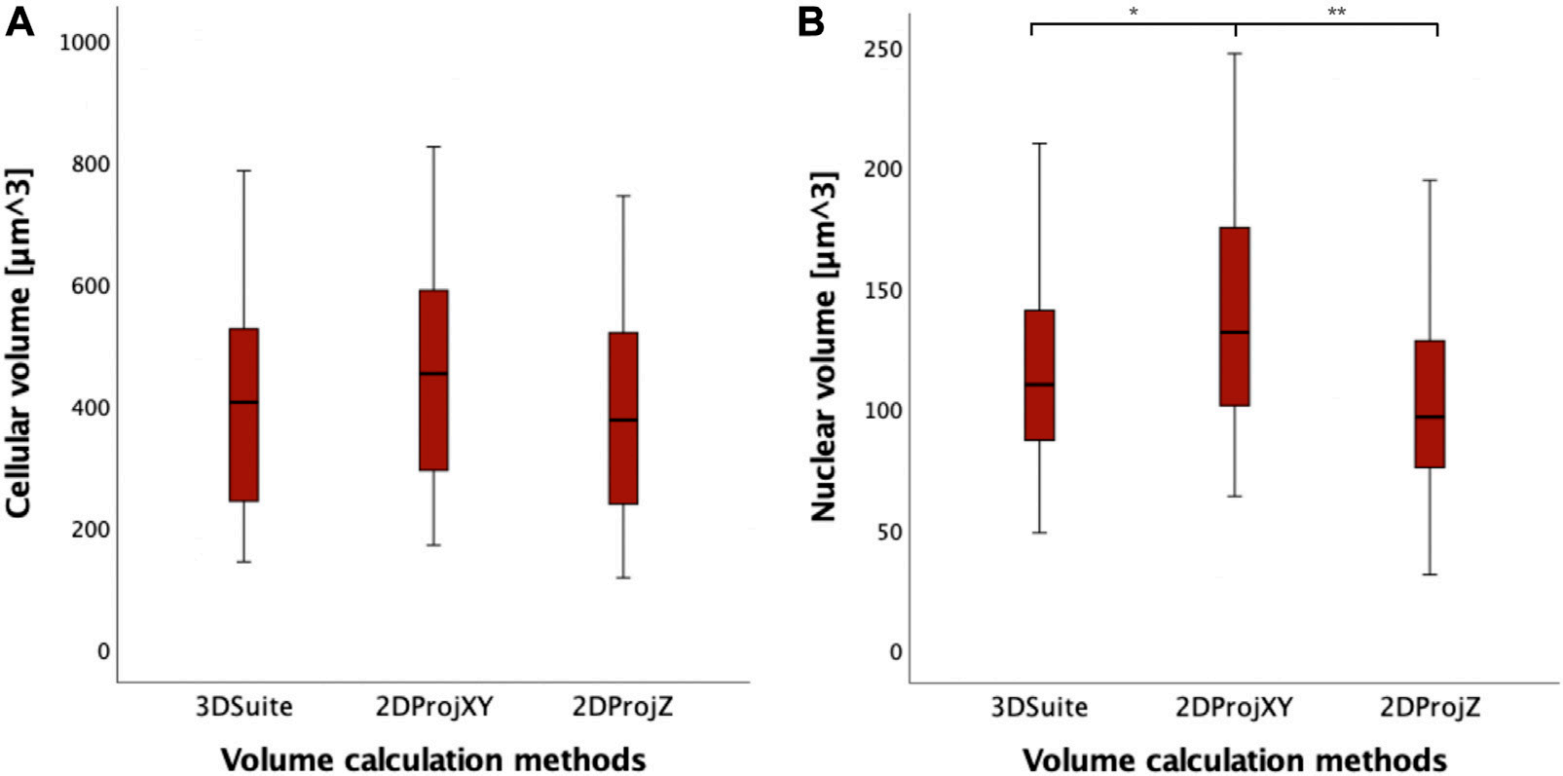
**(A)** Boxplots of cell volumes expressed in µm^3^, calculated with different methods (3DSuite, 2DProjXY, and 2DProjZ). **(B)** boxplot of nucleus volumes expressed in µm^3^, calculated with different methods (3DSuite, 2DProjXY, and 2DProjZ). The symbol * represents a statistically significant difference (*p < 0.05; **p < 0.01; ***p < 0.001).

Analyzing the distribution of data, 2DProjZ is the method with the lowest median volume value (403.80 µm^3^), 3DSuite is in the middle (422.78 µm^3^), and the 2DProjXY is the method with the highest median value (468.67 µm^3^) (Figure 7; Supplementary Table S6). The increase in volume in the 2DProjXY and partly in the 3DSuite appears related to elongation in the Z direction documented in Figure 7. However, no statistically significant difference in cell volumes has been observed between the three methods for calculating the volume of the 40 cells performing the non-parametric Mann-Whitney U Tests (*p* > 0.05). Considering instead the nuclear volume measurements computed by the three methods, a statistically significant increase is observed only in 2DProjXY compared both with 3DSuite (*p* = 0.02) and 2DProjZ (*p* = 0.001) (Supplementary Table S6).

**FIGURE 8:**
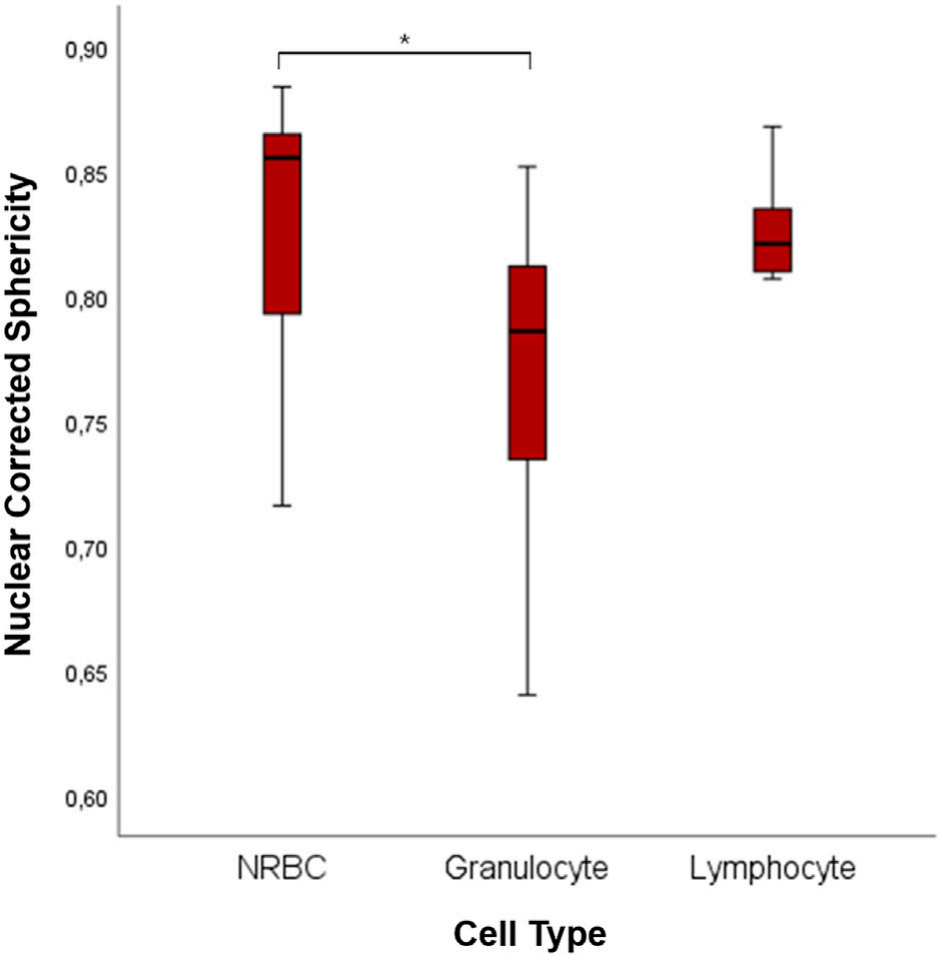
Boxplots of 3D nuclear sphericity values of NRBC, granulocyte, and lymphocyte. The symbol * represents a statistically significant difference (*p < 0.05; **p < 0.01; ***p < 0.001).

This difference can be related to the different shapes of the nuclei. Indeed, NRBCs and lymphocyte have spherical nuclei, whereas granulocytes have poly-lobated ones. We computed the nuclei sphericity to quantify such a difference (Figure 8). Granulocyte corrected nuclear sphericity has the lowest value, which is also statistically significantly different compared to NRBC (*p* = 0.011). However, the difference is not confirmed, from a statistical point of view, with respect to lymphocyte [NRBC: 0.855 (0.792–0.865); lymphocyte 0.821 (0.807–0.837); granulocyte 0.786 (0.729–0.818)].

## 4 Discussion

Tagless separation methods such as the FFF have been used to separate cells and microparticles. The separation is determined by several factors, such as cell and fluid density, viscosity, flow rate, cell dimensions, shape, and deformability (Guo et al., 2016). In this work, we analyzed the cell dimensions with a particular focus on blood cells, especially cord blood cells. To our knowledge, this is the first work to analyze the impact of two anticoagulants and two different fixative mixtures on the 2D and 3D dimensions and morphology of cells and nuclei. As mentioned, data on these cells are often obtained from fixed samples, whereas the cells are usually kept in physiological-like conditions during FFF. However, adding an anticoagulant to blood is the *conditio sine qua non* it is impossible to study blood cells. We, therefore, analyze how four different conditions (EDTA, CIT, CR, CS) affect cord blood cell dimensions, shape, and numerosity.

First of all, the cell size values we measured with the confocal microscopy are similar to those found in the literature (acquired through light microscopy, flow cytometry, and Coulter counter) for fresh and fixed blood cells (Schmid-Schönbein et al., 1980; Downey et al., 1985; Ruban et al., 2007; Makhro et al., 2016). Fixed granulocyte and lymphocyte represent an exception, as they are reported to be slightly larger in literature data (Löffler et al., 2005; Bain et al., 2015; Turgeon, 2018). Such a comparison supports our results, considering that the few differences might be related to the different measuring protocols (e.g., different fixatives).

We then start comparing data obtained with the two different anticoagulants. No statistically significant difference was found when comparing cell and nucleus diameter (EDTA vs. CIT) except in monocyte diameter, which was increased in the presence of citrate. One possible explanation for this phenomenon is that citrate in monocyte modulates the inflammatory response and could trigger differentiation into macrophage (Williams and O’Neill, 2018; Liu et al., 2021). Comprehensively, we can claim that cord blood cells have similar dimensions, shape, and numerosity when treated with EDTA or CIT, with few exceptions. Therefore, we can use them alternatively in future experiments unless the focus concerns the few differences highlighted above.

Then, we compared conditions with and without cell preservatives (EDTA and CIT vs. CR and CS). We point out that, from our data, the more cytoplasm is present in the cell, the more its size will be reduced by the fixation process. For instance, white blood cells have lower cytoplasm content than NRBCs, which are significantly reduced by fixation.

Additionally, we remark that the two fixation treatments differ in the amount of cell preservative in the solution. Indeed, cell preservative concentration directly affects cell membrane properties (Abay et al., 2019). Our data shows that red and white blood cells behave differently in the presence of CR or CS, namely, a different concentration of cell preservative.

Likely, these changes can be attributed to the different membrane and cytoskeleton characteristics and how they react to the fixation process. For instance, in the presence of CR, red blood cells’ diameter decreases compared to the EDTA and CIT cases, whereas white blood cells generally increase their diameter.

Finally, regarding cell diameter, we highlight the importance of osmosis when measuring cells with this technique, especially for the not-fixed samples. For this reason, we kept the samples wet with PBS, which has osmolarity comparable to plasma. Given that the fixing solution has a slightly greater osmolality, it might be responsible for water removal from the cells and the consequent diameter decrease during the fixation process. As known, in the presence of a fixative, the cell dehydrates. However, once resuspended in PBS for confocal analysis, the membrane fixed with a lower percentage of fixative remains less rigid and allows easier passage of water to restore osmolality. At higher concentrations, the membrane stiffness blocks the osmotic phenomenon (Kim et al., 2017). Probably in the presence of 3% fixative, the nuclear membrane swells more in the presence of PBS because it undergoes less cross-linking than the cell membrane, which is more exposed to fixatives. This claim is invalid in NRBC, in which the nucleus tends to be pycnotic and thus already at the highest degree of condensation.

We further analyze the cell shape and possible alterations when using cell preservatives. The shape descriptors are consistent across many cells and nucleus parameters. As also reported by Dinčić and colleagues (Dinčić et al., 2021), results in terms of circularity, solidity, and AR were coherent, assuming values near to one (Figure 4). Therefore, the circular shape of the cells is preserved in the four different conditions, depicting the absence of deformation in the cell structure in the presence of cell preservatives. On the other hand, the nucleus-to-cell ratio seems globally coherent with literature data when considering our fixed dataset (Turgeon, 2018; Dinčić et al., 2021), with few differences in which we identify lower ratios compared to the literature (e.g., lymphocyte).

Finally, regardless of the type of anticoagulant or fixative, no differences in cell counts were observed. Therefore, all four conditions perform equally in preserving the sample avoiding cell degradation and lysis.

Till this point, the entire analysis has been based on 2D images. However, we know from literature data that some structures have a non-spherical shape (e.g., poly-lobate nuclei). Therefore, we also considered 3D analysis on the confocal images to estimate cell volumes directly. As we documented, confocal microscopy might suffer from image distortion at this scale. The cause of this phenomenon has been identified in the refractive index mismatch between the immersion medium and the sample medium (Katiyar et al., 2021). Such a phenomenon generates artificially elongated images along the vertical direction (z), deforming the xz and yz cross-section profiles (Figure 6). Therefore, we compared the 2D and 3D analyses to evaluate how much such distortion can be seen in our data and how representative the 2D analysis is considering the different cell types. However, median differences in the cellular volumes among the three methods (3D, vertical projection, and lateral projections) are about 12% and 6%. For this reason, we based all the previously mentioned analysis on the 2D projection, which is not affected by this distortion (Diaspro et al., 2002; Kuypers et al., 2005; Van Elburg and Dirckx, 2007; Diel et al., 2020; Katiyar et al., 2021).

Besides that, the 3D analysis allows us to study the nuclei’s shape better. Indeed, using corrected nuclear sphericity, we distinguished the rounder nuclei of NRBC from the poly-lobate nuclei of granulocyte.

Not all of the granulocytes analyzed had an advanced stage of maturation, with a more poly-lobated nucleus, given the cord blood source of our samples. Some of them were in slightly more immature forms, with less segmentations. Such maturation spread causes a broader range of nuclear corrected sphericity with an overlap zone with the more homogeneous lymphocyte. Consequently, we report no statistically significant difference between these two cell populations. However, the 2D analysis is not enough to accurately estimate the granulocyte nucleus’s dimension, which might be more accurately computed with a complete 3D analysis. To this aim, 3D distortion must be avoided, following techniques similar to those reported by Heine and colleagues (Heine et al., 2018; Simionato et al., 2021).

## 5 Conclusion

This study used confocal microscopy *via* bio-imaging analysis to compare the size of umbilical cord blood cells in the presence of two anticoagulants and two fixative mixtures. Cord blood cells are affected by the different solutions we use to treat the samples, particularly considering the presence of cell preservatives. The fixation process appears to alter mainly cell dimension, especially in those cells with higher cytoplasm content. Additionally, the different cell preservative concentrations impact cell size differently. Therefore, choosing the solution to collect or fix the samples is crucial when analyzing the cell dimensions.

We also identify cases in which a 3D analysis can add information on cells’ shape and dimensions, mainly referring to cells’ nuclei. Those cases are characterized by non-spherical shapes, for which a 2D projection is insufficient to estimate their volume.

This information set is complemented with the estimate of relevant cell diameters in different conditions. Overall, these represent essential points for applying methods relying on cell diameter, such as GrFFF. In addition, even if this is an opinion of the authors, we believe that these results can probably be extended to blood cells of pathophysiological conditions (e.g., storage conditions in blood banks, hemoglobinopathies). However, further data are indeed required to verify this claim.

## Data Availability

The raw data supporting the conclusion of this article will be made available by the authors, without undue reservation.
