# Supplementary material for: Human umbilical cord blood cells suffer major modification by fixatives and anticoagulants
Source: Front Physiol. 2023 Mar 15;14:1070474. doi: 10.3389/fphys.2023.1070474 (PMC10050555; doi:10.3389/fphys.2023.1070474)
Supplement: Supplementary file 1 [file DataSheet1.doc]

**SUPPLEMENTARY**

The output of the Leica software platform, namely the .lif file containing the metadata about the acquired images, was imported into ImageJ. The file was read by Bio-Format Importer, a specific Fiji plugin [Linkert_2010].


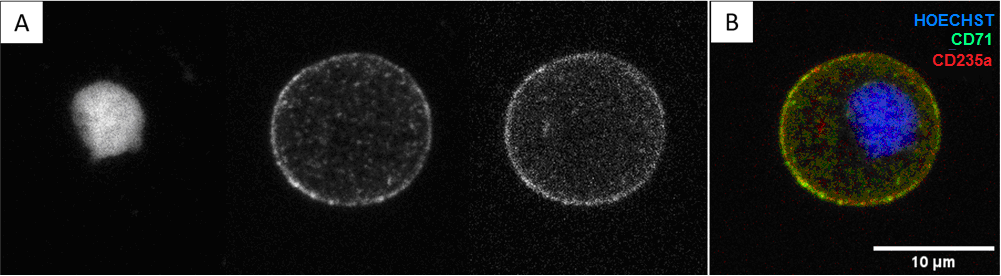


***Figure S1***: *Panel A) is the original 8-bit image, composed of the sum of the stack image slices along the z-axis of each channel: the left one is the nucleus, i.e. Hoechst, and the middle and right ones are the channels of the same cell, i.e. CD71 and CD235a, respectively. Panel B) is the RGB image with the corresponding scale.*

 
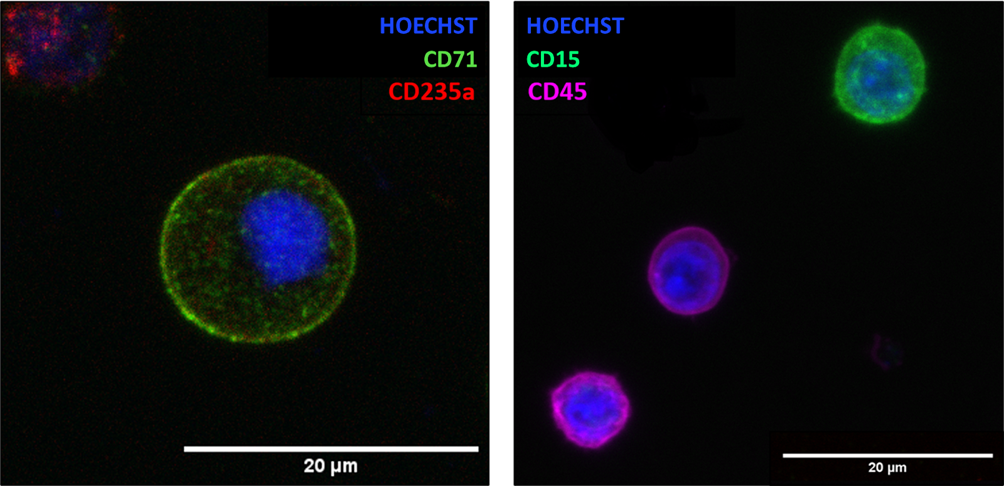


***Figure S2****: The panels show the results of the first section of the ImageJ macro: the RGB images. The left panel shows a CD45- fresh cord blood sample, specifically the cell is an orthochromatic erythroblast, while the right panel shows a CD45+ fresh cord blood sample with two lymphocytes (stained in magenta) and a granulocyte (stained in green).*

1. Editing of RGB image:

a.              "Image > Type > 8-Bit": greyscale conversion;

b.              "Edit > Invert": creates a reversed image of the entire stack;

c.              "Image > Stacks > Z Project. . . ": choosing Sum Slices as Projection Type, overlaps all the sections in the stack along the z-axis;

d.               “Image > Colour > Split Channels”: divides the summed slices, in the previous point, as a series of single images equal to the number of channels chosen during the acquisition phase (in this case, they are 3 channels) (Figure S1, panel A) );

e.              “Image > Colour > Merge Channels”: builds the RGB image, merging the previously split channel's image into a single coloured one, according to the analysed sample type (namely, the presence or not of the CD45) (Figure S1, panel B) ).

 
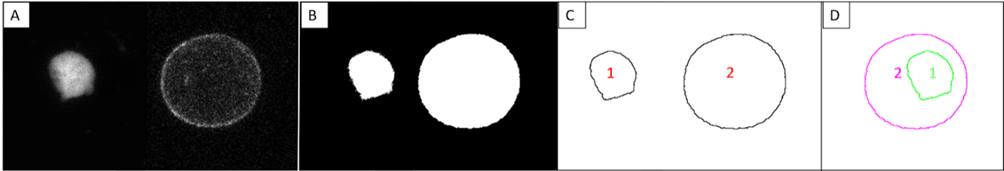


***Figure S3****: A) Original image; B) Manually thresholded image; C) Contours of the nucleus (left one, labelled with "1") and the cell (right one, labelled with "2"); D) Merging of the previous drawings, where the labels are the same of the C) panel.*

2. Computing particle analysis for both nucleus and cell:

a.              “Image > Adjust > Brightness/Contrast…” and “Image > Adjust > Threshold“: convert the image into a binary one, setting the threshold manually, a mandatory step to measure cells diameters (Figure S3, panel B));

b.              The image pre-processing phase consists of:

a.              “Process > Binary > Fill Holes”: fills holes in objects by filling the background;

b.              “Process > Noise > Despeckle”: removes noise and preserves boundaries better than simple average filtering;

c.              “Process > Binary > Watershed”: splits adjacent objects;

c.              “Analyze > Analyze Particles…” is the main command to obtain the result data, that are the essential core of this study: dimension information (area, measured in µm2), the position of centroid, major and minor diagonal, shape descriptors as circularity, roundness and solidity (as reported in Table S1) and the contour outline as visualization method of the cell and the nucleus (Figure S3, panel C) ).

d.              “Process > Image Calculator…”: overlies the contours of the cell and the nucleus as reported in Figure S3, panel D).

***Table S1****: Results table as the output of “Analyze Particles…” ImageJ command. The table is referring to Figure S3*~~.~~

|  | **Area [µm^2]** | **X** | **Y** | **Perim. [µm]** | **Major [µm]** | **Minor [µm]** | **Angle** | **Circ [-]** | **AR   [-]** | **Solidity [-]** |
| --- | --- | --- | --- | --- | --- | --- | --- | --- | --- | --- |
| **1** | 29.739 | 14.294 | 9.793 | 21.844 | 6.604 | 5.734 | 119.165 | 0.783 | 1.152 | 0.957 |
| **2** | 137.379 | 12.419 | 10.733 | 44.773 | 13.617 | 12.846 | 11.504 | 0.861 | 1.060 | 0.983 |

***Table S2:*** *p-values from non-parametric Mann-Whitney U Tests of cell diameters, nucleus diameters and nucleus percentage. A cross-comparison among all the samples has been conducted.*

| **Cell Diameter** | | | | | | | | | | | | | | | | | | |
| --- | --- | --- | --- | --- | --- | --- | --- | --- | --- | --- | --- | --- | --- | --- | --- | --- | --- | --- |
|  | NRBC | | | Granulocyte | | | Lymphocyte | | | Monocyte | | | RBC | | | | | |
| P values | CIT | CR | CS | CIT | CR | CS | CIT | CR | CS | CIT | CR | CS | CIT | | CR | CS | | |
| EDTA | 0,921 | <0,001 | <0,001 | 0,236 | <0,001 | 0,100 | 0,231 | <0,001 | 0,431 | 0,017 | 0,001 | 0,388 | 0,770 | | 0,021 | 0,002 | | |
| CIT |  | <0,001 | 0,002 |  | <0,001 | 0,007 |  | <0,001 | 0,079 |  | 0,182 | 0,001 |  | | 0,027 | 0,001 | | |
| CR |  |  | 0,013 |  |  | <0,001 |  |  | <0,001 |  |  | 0,001 |  | |  | 0,723 | | |
| **Nucleus Diameter** | | | | | | | | | | | | | |  | | |  |  |
|  | NRBC | | | Granulocyte | | | Lymphocyte | | | Monocyte | | | |  | | |  |  |
| P values | CIT | CR | CS | CIT | CR | CS | CIT | CR | CS | CIT | CR | CS | |  | | |  |  |
| EDTA | 0.434 | 0.187 | 0.168 | 0.297 | <0,001 | 0.686 | 0.471 | <0,001 | 0.976 | 0.179 | 0.019 | 0.177 | |  | | |  |  |
| CIT |  | 0.667 | 0.027 |  | <0,001 | 0.428 |  | <0,001 | 0.429 |  | 0.002 | 0.901 | |  | | |  |  |
| CR |  |  | 0.001 |  |  | <0,001 |  |  | <0,001 |  |  | 0.003 | |  | | |  |  |
| **Nucleus percentage** | | | | | | | | | | | | | |  | | |  |  |
|  | NRBC | | | Granulocyte | | | Lymphocyte | | | Monocyte | | | |  | | |  |  |
| P values | CIT | CR | CS | CIT | CR | CS | CIT | CR | CS | CIT | CR | CS | |  | | |  |  |
| EDTA | 0,732 | <0,001 | 0,018 | 0,001 | 0,157 | 0,268 | 0,072 | <0,001 | 0,081 | <0,001 | 0,325 | 0,777 | |  | | |  |  |
| CIT |  | <0,001 | 0,140 |  | 0,060 | <0,001 |  | <0,001 | 0,004 |  | 0,047 | 0,011 | |  | | |  |  |
| CR |  |  | <0,001 |  |  | 0,026 |  |  | <0,001 |  |  | 0,548 | |  | | |  |  |

***Table S3:*** *p-values highlighting significance of the differences among cell populations (N=NRBC; G=Granulocyte; L=Lymphocyte; M=Monocyte) regarding different shape descriptors under the four conditions. p-values lower than 0,001 are shown in dark blue, lower than 0,01 in blue, and lower that 0,05 in light blue. Note the different distribution of the significant differences across the four conditions, showing how the anticoagulant and the preservatives impact the cell-related differences.*

|  | **Cellular Aspect Ratio** | | | | | | | | | | | | | | | |
| --- | --- | --- | --- | --- | --- | --- | --- | --- | --- | --- | --- | --- | --- | --- | --- | --- |
|  | EDTA | | | | CIT | | | | CR | | | | CS | | | |
|  | N | G | L | M | N | G | L | M | N | G | L | M | N | G | L | M |
| RBC | 0,322 | 0,957 | 0,349 | 0,384 | 0,809 | 0,02 | 0,616 | 0,288 | 0,049 | <0,001 | <0,001 | 0,075 | 0,963 | <0,001 | 0,841 | 0,758 |
| NRBC |  | 0,124 | 0,002 | 0,744 |  | 0,007 | 0,711 | 0,242 |  | 0,004 | 0,004 | 0,917 |  | 0,001 | 0,874 | 0,872 |
| Granulocyte |  |  | 0,054 | 0,166 |  |  | 0,007 | 0,05 |  |  | 0,977 | 0,019 |  |  | <0,001 | 0,004 |
| Lymphocyte |  |  |  | 0,001 |  |  |  | 0,401 |  |  |  | 0,032 |  |  |  | 0,609 |
|  | **Cellular Circularity** | | | | | | | | | | | | | | | |
|  | EDTA | | | | CIT | | | | CR | | | | CS | | | |
|  | N | G | L | M | N | G | L | M | N | G | L | M | N | G | L | M |
| RBC | 0,806 | <0,001 | 0,018 | <0,001 | 0,026 | <0,001 | <0,001 | <0,001 | 0,136 | 0,003 | 0,006 | <0,001 | <0,001 | <0,001 | 0,176 | <0,001 |
| NRBC |  | <0,001 | <0,001 | <0,001 |  | 0,015 | 0,078 | <0,001 |  | <0,001 | <0,001 | <0,001 |  | 0,001 | 0,015 | <0,001 |
| Granulocyte |  |  | <0,001 | <0,001 |  |  | 0,37 | <0,001 |  |  | 0,722 | <0,001 |  |  | <0,001 | 0,009 |
| Lymphocyte |  |  |  | <0,001 |  |  |  | <0,001 |  |  |  | <0,001 |  |  |  | <0,001 |
|  | **Cellular Solidity** | | | | | | | | | | | | | | | |
|  | EDTA | | | | CIT | | | | CR | | | | CS | | | |
|  | N | G | L | M | N | G | L | M | N | G | L | M | N | G | L | M |
| RBC | 0,038 | <0,001 | 0,004 | <0,001 | 0,178 | <0,001 | <0,001 | <0,001 | 0,08 | 0,574 | 0,043 | <0,001 | 0,367 | <0,001 | 0,961 | <0,001 |
| NRBC |  | <0,001 | <0,001 | <0,001 |  | 0,028 | 0,002 | <0,001 |  | 0,002 | <0,001 | <0,001 |  | 0,002 | 0,232 | <0,001 |
| Granulocyte |  |  | 0,007 | <0,001 |  |  | 0,403 | <0,001 |  |  | 0,143 | <0,001 |  |  | <0,001 | 0,015 |
| Lymphocyte |  |  |  | <0,001 |  |  |  | <0,001 |  |  |  | <0,001 |  |  |  | <0,001 |

***Table S4:*** p-values highlighting significance of the differences among the nuclei regarding different shape descriptors under the four conditions (N=NRBC; G=Granulocyte; L=Lymphocyte; M=Monocyte). p-values lower than 0,001 are shown in dark blue, lower than 0,01 in blue, and lower that 0,05 in light blue. Note the different distribution of the significant differences across the four conditions, showing how the anticoagulant and the preservatives impact the nuclei-related differences.

|  | **Nuclear Aspect Ratio** | | | | | | | | | | | |
| --- | --- | --- | --- | --- | --- | --- | --- | --- | --- | --- | --- | --- |
|  | EDTA | | | CIT | | | CR | | | CS | | |
|  | G | L | M | G | L | M | G | L | M | G | L | M |
| NRBC | <0,001 | <0,001 | 0,705 | <0,001 | 0,153 | 0,329 | <0,001 | <0,001 | <0,001 | <0,001 | <0,001 | <0,001 |
| Granulocyte |  | <0,001 | <0,001 |  | <0,001 | <0,001 |  | <0,001 | 0,044 |  | <0,001 | 0,063 |
| Lymphocyte |  |  | 0,003 |  |  | 0,005 |  |  | 0,008 |  |  | 0,01 |
|  | **Nuclear Circularity** | | | | | | | | | | | |
|  | EDTA | | | CIT | | | CR | | | CS | | |
|  | G | L | M | G | L | M | G | L | M | G | L | M |
| NRBC | <0,001 | 0,034 | <0,001 | 0,022 | 0,024 | <0,001 | <0,001 | <0,001 | <0,001 | <0,001 | <0,001 | <0,001 |
| Granulocyte |  | <0,001 | 0,051 |  | 0,827 | 0,003 |  | 0,001 | 0,175 |  | <0,001 | <0,001 |
| Lymphocyte |  |  | <0,001 |  |  | <0,001 |  |  | <0,001 |  |  | 0,072 |
|  | **Nuclear Solidity** | | | | | | | | | | | |
|  | EDTA | | | CIT | | | CR | | | CS | | |
|  | G | L | M | G | L | M | G | L | M | G | L | M |
| NRBC | <0,001 | 0,009 | <0,001 | 0,091 | 0,004 | <0,001 | <0,001 | <0,001 | <0,001 | <0,001 | <0,001 | 0,032 |
| Granulocyte |  | <0,001 | 0,207 |  | 0,553 | 0,002 |  | 0,008 | 0,246 |  | <0,001 | <0,001 |
| Lymphocyte |  |  | <0,001 |  |  | 0,001 |  |  | <0,001 |  |  | 0,857 |

***Table S5:*** *p-values highlighting significance of the differences among the tubes regarding different cellular shape descriptors of the cells. p-values lower than 0,001 are shown in dark blue, lower than 0,01 in blue, and lower that 0,05 in light blue.*

| **Cellular Aspect Ratio** | | | | | | | | | | | | | | | |
| --- | --- | --- | --- | --- | --- | --- | --- | --- | --- | --- | --- | --- | --- | --- | --- |
|  | NRBC | | | RBC | | | Granulocyte | | | Lymphocyte | | | Monocyte | | |
|  | EDTA | CIT | CS | EDTA | CIT | CS | EDTA | CIT | CS | EDTA | CIT | CS | EDTA | CIT | CS |
| CIT | 0,070 |  |  | 0,598 |  |  | 0,010 |  |  | 0,394 |  |  | 0,512 |  |  |
| CS | 0,066 | 0,831 |  | 0,666 | 0,721 |  | <0,001 | 0,980 |  | 0,699 | 0,675 |  | 0,160 | 0,506 |  |
| CR | <0,001 | 0,346 | 0,210 | 0,013 | 0,066 | 0,005 | 0,320 | 0,192 | 0,126 | 0,021 | 0,163 | 0,075 | 0,003 | 0,059 | 0,187 |
| **Cellular Circularity** | | | | | | | | | | | | | | | |
|  | NRBC | | | RBC | | | Granulocyte | | | Lymphocyte | | | Monocyte | | |
|  | EDTA | CIT | CS | EDTA | CIT | CS | EDTA | CIT | CS | EDTA | CIT | CS | EDTA | CIT | CS |
| CIT | <0,001 |  |  | 0,934 |  |  | 0,750 |  |  | <0,001 |  |  | <0,001 |  |  |
| CS | 0,401 | <0,001 |  | 0,031 | 0,109 |  | 0,116 | 0,123 |  | <0,001 | <0,001 |  | 0,671 | 0,008 |  |
| CR | 0,110 | 0,073 | <0,001 | 0,627 | 0,633 | 0,013 | 0,002 | <0,001 | 0,089 | 0,077 | <0,001 | <0,001 | <0,001 | 0,414 | 0,009 |
| **Cellular Solidity** | | | | | | | | | | | | | | | |
|  | NRBC | | | RBC | | | Granulocyte | | | Lymphocyte | | | Monocyte | | |
|  | EDTA | CIT | CS | EDTA | CIT | CS | EDTA | CIT | CS | EDTA | CIT | CS | EDTA | CIT | CS |
| CIT | 0,008 |  |  | 0,321 |  |  | 0,700 |  |  | 0,005 |  |  | <0,001 |  |  |
| CS | 0,001 | 0,916 |  | 0,898 | 0,208 |  | 0,141 | 0,408 |  | <0,001 | <0,001 |  | 0,490 | 0,004 |  |
| CR | 0,426 | 0,044 | 0,008 | 0,627 | 0,264 | 0,387 | 0,025 | 0,071 | 0,175 | 0,408 | 0,131 | <0,001 | 0,009 | 0,89 | 0,021 |

***Table S6:*** *p-values highlighting significance of the differences among the tubes regarding different nuclear shape descriptors of the cells. p-values lower than 0,001 are shown in dark blue, lower than 0,01 in blue, and lower that 0,05 in light blue.*

| **Nuclear Aspect Ratio** | | | | | | | | | | | | |
| --- | --- | --- | --- | --- | --- | --- | --- | --- | --- | --- | --- | --- |
|  | NRBC | | | Granulocyte | | | Lymphocyte | | | Monocyte | | |
|  | EDTA | CIT | CS | EDTA | CIT | CS | EDTA | CIT | CS | EDTA | CIT | CS |
| CIT | 0,235 |  |  | 0,114 |  |  | 0,489 |  |  | 0,935 |  |  |
| CS | <0,001 | <0,001 |  | 0,672 | 0,310 |  | 0,696 | 0,320 |  | 0,319 | 0,349 |  |
| CR | <0,001 | <0,001 | 0,030 | 0,045 | 0,560 | 0,130 | 0,687 | 0,420 | 0,950 | 0,121 | 0,140 | 0,570 |
| **Nuclear Circularity** | | | | | | | | | | | | |
|  | NRBC | | | Granulocyte | | | Lymphocyte | | | Monocyte | | |
|  | EDTA | CIT | CS | EDTA | CIT | CS | EDTA | CIT | CS | EDTA | CIT | CS |
| CIT | 0,623 |  |  | 0,009 |  |  | 0,351 |  |  | 0,540 |  |  |
| CS | <0,001 | 0,003 |  | <0,001 | <0,001 |  | 0,010 | 0,009 |  | <0,001 | 0,002 |  |
| CR | <0,001 | <0,001 | <0,001 | 0,150 | 0,376 | <0,001 | 0,025 | 0,021 | 0,523 | 0,623 | 0,838 | 0,014 |
| **Nuclear Solidity** | | | | | | | | | | | | |
|  | NRBC | | | Granulocyte | | | Lymphocyte | | | Monocyte | | |
|  | EDTA | CIT | CS | EDTA | CIT | CS | EDTA | CIT | CS | EDTA | CIT | CS |
| CIT | 0,116 |  |  | 0,003 |  |  | 0,940 |  |  | 0,770 |  |  |
| CS | <0,001 | 0,228 |  | <0,001 | <0,001 |  | 0,006 | 0,033 |  | <0,001 | <0,001 |  |
| CR | <0,001 | <0,001 | <0,001 | 0,005 | 0,936 | <0,001 | <0,001 | <0,001 | 0,003 | 0,076 | 0,197 | 0,089 |

***Table S7****: Median (1st - 3rd quartile) cellular and nuclear volumes measured in the three different methods (3DSuite, 2DProjXY, 2DProjZ) and percentage of differences between 2 compared methods. The symbol §, indicates that the data are normally distributed.*

| **Volume [µm^3]** | **N** | **3DSuite** | **2DProjXY** | **2DProjZ** |
| --- | --- | --- | --- | --- |
| **Cells** | 40 | 422.78 (285.85 - 512.17)*§* | 468.67 (328.2 - 587.63)*§* | 403.80 (251.49 - 516.77)*§* |
| **Cellular Sphericity** |  | 0.856 (0.835 - 0.862) |  |  |
| **Nuclei** | 40 | 116.60 (89.16 - 140.92)*§* | 133.83 (105.42 - 178.26)*§* | 97.70 (82.80 - 129.93)*§* |
| **Nuclear Sphericity** |  | 0.817 (0.785 - 0.851) |  |  |
| **%Differences** |  | **3DSuite vs 2DProjXY** | **3DSuite vs 2DProjZ** | **2DProjXY vs 2DProjZ** |
| **Cells** | 40 | -12.38 (-18.55 - -9.6) | 5.84 (3.34 - 10.54) | -17.56 (-29.87 - -13.20) |
| **Nuclei** |  | -19.49 (-30.11 - -15.28) | 8.88 (0.2 - 21.33) | -41.67 (-60.6 - -24.18) |
